# Highly Sensitive Ethanol Chemical Sensor Based on Novel Ag-Doped Mesoporous α–Fe_2_O_3_ Prepared by Modified Sol-Gel Process

**DOI:** 10.1186/s11671-018-2572-8

**Published:** 2018-05-21

**Authors:** Moteb M. Alqahtani, Atif M. Ali, Farid A. Harraz, M. Faisal, Adel A. Ismail, Mahmoud A. Sayed, M. S. Al-Assiri

**Affiliations:** 1 Department of Physics, Faculty of Science, King Khalid University, Abha, Saudi Arabia; 2 Department of Physics, Faculty of Science, Assiut University, Assiut, Egypt; 3 Promising Centre for Sensors and Electronic Devices (PCSED), Advanced Materials and Nano-Research Centre, Najran University, P.O. Box: 1988, Najran, 11001 Saudi Arabia; 4 Nanomaterials and Nanotechnology Department, Central Metallurgical Research and Development Institute (CMRDI), P.O. 87, Helwan, Cairo 11421 Egypt; 5 Department of Physics, Faculty of Science and Arts, Najran University, Najran, Saudi Arabia

**Keywords:** Mesoporous Ag/α–Fe_2_O_3_, Sol-gel, Electrochemical, Chemical sensors, Ethanol

## Abstract

Mesoporous α–Fe_2_O_3_ has been synthesized via a simple sol-gel procedure in the presence of Pluronic (F-127) triblock copolymer as structure directing agent. Silver (Ag) nanoparticles were deposited onto α–Fe_2_O_3_ matrix by the photochemical reduction approach. Morphological analysis revealed the formation of Ag nanoparticles with small sizes < 20 nm onto the mesoporous structure of α–Fe_2_O_3_ possessing < 50 nm semi-spherical shape. The XRD, FTIR, Raman, UV-vis, PL, and N_2_ sorption isotherm studies confirmed the high crystallinity, mesoporosity, and optical characteristics of the synthesized product. The electrochemical sensing toward liquid ethanol has been performed using the current devolved Ag/α–Fe_2_O_3_-modified glassy carbon electrode (GCE) by cyclic voltammetry (*CV*) and current potential (*I-V*) techniques, and the obtained results were compared with bare GCE or pure α–Fe_2_O_3_. Mesoporous Ag/α–Fe_2_O_3_ was found to largely enhance the sensor sensitivity and it exhibited excellent sensing characteristics during the precision detection of low concentrations of ethanol. High and reproducible sensitivity of 41.27 μAmM^− 1^ cm^− 2^ at lower ethanol concentration region (0.05 to 0.8 mM) and 2.93 μAmM^− 1^ cm^− 2^ at higher concentration zone (0.8 to 15 mM), with a limit of detection (LOD) of 15.4 μM have been achieved. Investigation on reaction kinetics revealed a characteristic behavior of mixed surface and diffusion-controlled processes. Detailed sensing studies revealed also that the sensitivity toward ethanol was higher than that of methanol or isopropanol. With further effort in developing the synthesis and fabrication approaches, a proper utility for the current proposed protocol for fabricating a better sensor device performance is possible.

## Background

The research area of chemical sensors has expanded significantly in the past decade due to its importance in a vast range of technological applications in the fields of diagnostic and drug discovery, safety-related issues, food industries, environmental monitoring, and agricultural analyses [1, 2]. Based on the physical property to be determined, chemical sensors could be classified as optical, electrical, thermal, or mass sensors, and they are appropriate to detect target analytes either in gaseous, liquid, or solid state [3]. Among the presently available sensors, the electrochemical sensors are particularly attractive owing to remarkable sensitivity, expected fast response time, simplicity of experimental set-up and lower cost [4]. In electrochemical sensors, the working electrodes are essentially modified with the active sensing materials. The physico-chemical properties of active materials affect greatly the sensor performance as well as its operational stability [5]. Therefore, the research and development for a potential active material play a decisive role in fabricating sensitive, efficient, and reliable sensing devices. Moreover, with the aid of nanotechnology, it is now likely to synthesize a wide range of novel nanomaterials with specific shapes and morphologies, which could lead to unique physico-chemical characteristics [6–8]. Particularly, metal oxide semiconductors are unique class of nano-materials that have been received considerable attention because of their promising sensing performances as they could promote the electron-transfer kinetics [9–13], in addition to their attractive characteristics such as ease of fabrication, ability to control size and morphology, ease to modify surface, good chemical stability and catalytic properties [14]. They showed also strong affinity toward the adsorption of target molecules [15–18]. Various types of metal oxide semiconductors have been successfully synthesized with different morphologies; nanoparticles, nanowires, nanorods, nanotubes, nanosheets, nanobelts, and quantum dots using various synthetic routes such as hydrothermal/solvothermal [19–21], sol–gel [22, 23], growth in aqueous solutions [24], chemical deposition [25], electrochemical technique [26], and chemical and physical vapor deposition [27, 28]. However, development of novel, effective metal oxide semiconductors for chemical sensor applications is still an existing challenge that requires suitable manipulation and optimization of materials with a careful selection of appropriate working electrode.

As an *n*-type semiconductor, the α–Fe_2_O_3_ (hematite phase of iron oxides) is a notably promising oxide category characterized by high stability, corrosion resistance, nontoxicity, and has found a wide uses as gas and chemical sensing material [29–31], as pigments and in magnetic recording media, photocatalysis, and photoanode in water splitting [32–34]. For example, chemical sensor based on α–Fe_2_O_3_ nanoparticles has been fabricated with high resistance variation for the detection of CH_3_SH gas, at room temperature in the range of 20–80 ppm [35]. In another report, Ag-doped Fe_2_O_3_ as core-shell nanocomposites have shown a good sensitivity to NO_2_ gas and could detect as low as 0.5 ppm NO_2_ [36]. A tertiary nanocomposite of Ag–Fe_2_O_3_–rGO was also synthesized via chemical reduction and hydrothermal method and successfully employed as a non-enzymatic H_2_O_2_ sensor [37]. A nanocomposite of α–Fe_2_O_3_–GO with different Fe_2_O_3_ contents have been designed and used for enhanced sensing performance toward ethanol gas [38]. In this contribution, a novel Ag/α–Fe_2_O_3_ hybrid nanostructure has been synthesized through a simple, modified sol-gel procedure using Pluronic (F-127) triblock copolymer as structure directing agent followed by a photoreduction approach to deposit Ag nanoparticles. The newly developed mesoporous Ag/α–Fe_2_O_3_ has been explored the attractive properties of both components (noble metal nanoparticles and mesoporous metal oxide) as a sensitive chemical sensor to effectively detect liquid ethanol at low concentration via cyclic voltammetry and current-potential (*I-V*) techniques. To the best of our knowledge, the current proposed hybrid mesostructure has not been used before for the electrochemical detection of ethanol.

## Methods/Experimental

### Materials

The block copolymer surfactant EO_106_–PO_70_EO_106_ (F-127, EO **=** –CH_2_CH_2_O–, PO **=** –CH_2_(CH_3_)CHO–), MW 12600 g/mol), iron nitrate Fe(NO_3_)_3_.9H_2_O, ethanol C_2_H_5_OH, silver nitrate AgNO_3_ were purchased from Sigma-Aldrich and used as received without further purification.

### Synthesis of Mesoporous α–Fe_2_O_3_

Mesoporous α–Fe_2_O_3_ nanocrystals were synthesized via sol-gel procedure using F-127 as a template directing agent. The following molar ratios of starting precursors were employed: Fe(NO_3_)_3_.9H_2_O /F127/C_2_H_5_OH/HCl/CH_3_COOH = 1:0.02:50:2.25:3.75. In a typical synthetic run, 1.6 g of F127 was added to 30 mL ethanol with stirring until obtaining a clear solution. Then 2.3 mL CH_3_COOH, 0.74 mL HCl, and 4.4 g iron nitrate were subsequently added to the above solution with vigorously stirring for 60 min and finally transferred into a Petri dish for the gelation step. The as-synthesized mesophase was dried and aged at 40 °C and 40% humidity for 12 h followed by further aging at 65 °C for 24 h. A calcination step was performed and adapted at 450 °C for 4 h at a heating rate 1 °C/min and a cooling rate of 1 °C/min to obtain mesoporous α–Fe_2_O_3_ nanocrystals.

### Photochemical Reduction of Ag Ions onto Mesoporous α–Fe_2_O_3_

Ag was deposited onto mesoporous α–Fe_2_O_3_ by the photochemical reduction of silver ions according to the following procedure: a suspended solution containing 1.0 g mesoporous α–Fe_2_O_3_ and 9.4 × 10^− 5^ mol AgNO_3_ was sonicated in 100 mL aqueous methanol (1% (*v*/*v*) methanol/H_2_O). The solution was illuminated using a Philips Hg lamp UV(A) light (intensity = 2.0 mWcm^− 2^) for 12 h. The as-produced Ag/α–Fe_2_O_3_ was separated by centrifugation, washed with deionized water and ethanol, and dried at 110 °C for 12 h.

### Materials Characterization

X-ray diffraction patterns (XRD) were measured by a PANalytical X’ port diffractometer using Cu Kα_1/2_, λα_1_ = 154.060 pm, λα_2_ = 154.439 pm radiation. Fourier transforms infrared spectrometer (FT-IR) spectrum was collected in the range from 400 to 4000 cm^− 1^ using BRUKER FRA 106 spectrometer using the standard KBr pellet procedure. Raman spectra were measured using a Perkin Elmer Raman Station 400. UV-visible spectrophotometer (lambda 950 Perkin Elmer) was used for the UV-vis optical absorption spectra measurement at room temperature in the range 200–800 nm. Room temperature photoluminescence (PL) spectra were collected on spectrofluorophotometer, (RF-5301 PC, Japan, SHIMADZU, 400 W, 50/60 Hz) using a 150 W xenon lamp at 315 nm excitation wavelength. Surface morphology was investigated by field emission-secondary electron microscope (FE-SEM) with a FE scanning electron microanalyzer (JEOL-6300F, 5 kV), equipped with EDS analysis. Quantachrome NOVA Station A was used for obtaining nitrogen adsorption/desorption isotherms at 77 K for the samples vacuum-dried at 300 °C for 3 h. Barrett-Joyner-Halenda (BJH) model with Halsey equation were applied to calculate sorption data [39].

### Electrochemical Detection of Ethanol in Aqueous Solutions

**Scheme 1:**
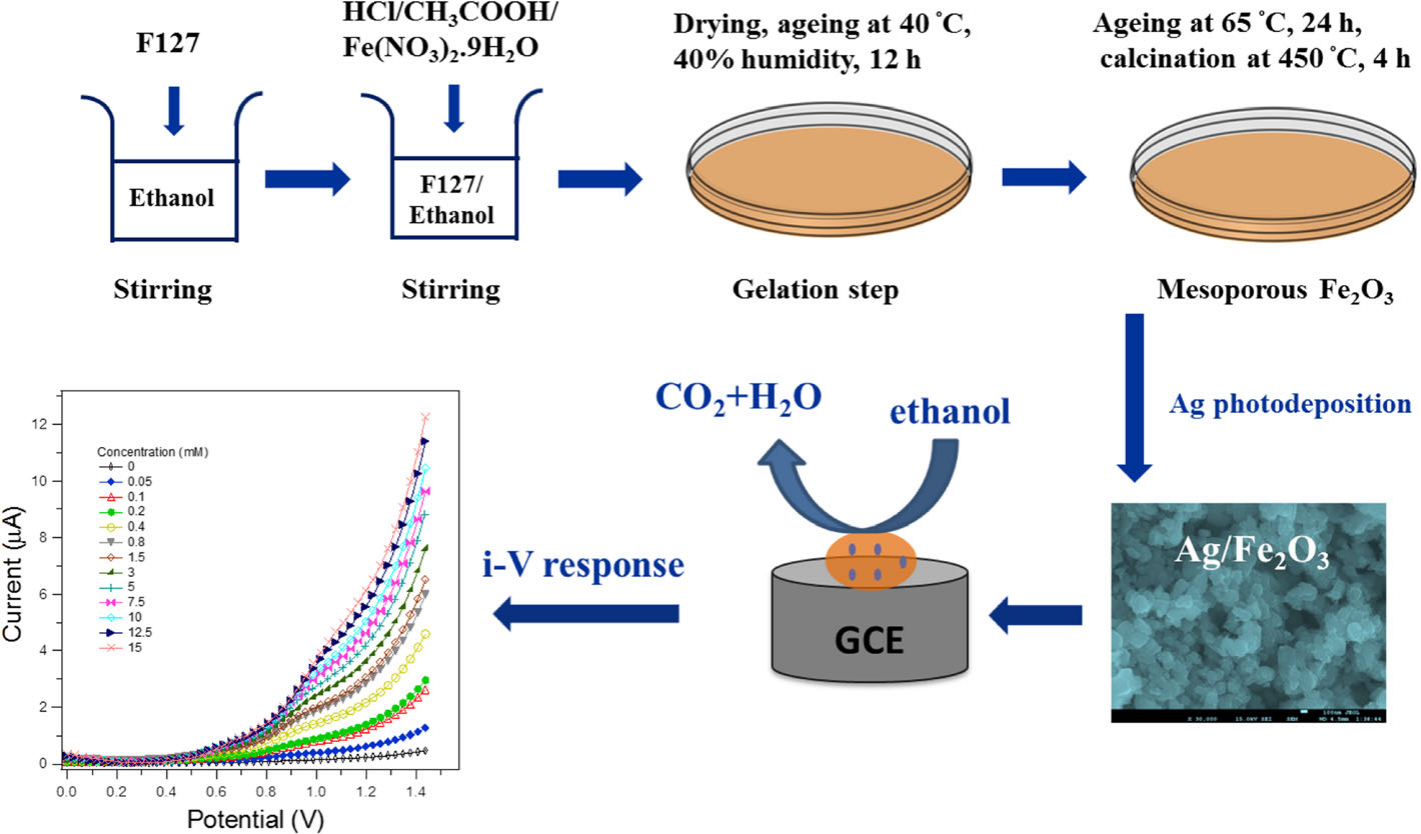
Schematic illustration of the synthesis of Ag/α–Fe_2_O_3_-modified GCE, along with the electrochemical detection of ethanol

Glassy carbon electrodes (GCE) with surface area 0.071 cm^2^ (Bio-Logic SAS) were initially polished with 1 and 0.05 μm polishing diamond and alumina slurry, respectively, washed with deionized water, sonicated in ethanol, water and finally left for naturally drying. The GCE was subsequently coated by Ag/α–Fe_2_O_3_ active material using a butyl carbitol acetate and ethyl acetate as conducting binders. The modified GCE was then dried overnight at 65 °C. A typical two electrode electrochemical cell with a working electrode (modified GCE) and a counter electrode (a Pt wire) was connected to the electrochemical work station, ZahnerZennium, Germany. A three electrode cell using Ag/AgCl reference electrode was also used for the cyclic voltammetry investigation. A 0.1 M concentration of PBS (phosphate buffer solution) of pH 7 was prepared from Na_2_HPO_4_ and NaH_2_PO_4_ and acted as a supporting electrolyte. Various ethanol concentrations ranging from 0.05 to 15 mM were applied in this study. The *I-V* (current-potential) characteristics were measured under continuous stirring, room temperature, in the anodic direction within a potential window from 0 to 1.5 V at 50 mV/s scan rate. The sensor sensitivity was estimated from the slope of the corresponding calibration curve of current versus ethanol concentration divided by the GCE surface area. The LOD (limit of detection) was calculated at a *S*/*N* = 3 (signal-to-noise ratio). A schematic illustration for the synthesis of Ag/α–Fe_2_O_3_ with the electrochemical detection of ethanol is depicted in Scheme 1.

## Results and Discussion

### Structural, Optical, and Morphological Investigation of Mesoporous Ag/α–Fe_2_O_3_

**Fig. 1:**
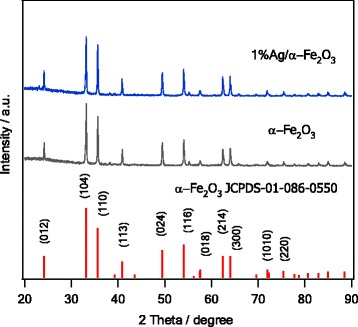
XRD patterns of as-synthesized α−Fe_2_O_3_ and 1%Ag/α−Fe_2_O_3_. The standard pattern of pure α−Fe_2_O_3_ is also shown

The phase and crystallinity of as-synthesized materials were firstly examined by XRD. As could be revealed from Fig. 1, the XRD spectrum of the sol-gel derived α–Fe_2_O_3_ is consistent with the standard pattern of pure α − Fe_2_O_3_. All peaks can be assigned perfectly to the crystalline phase of α–Fe_2_O_3_, (JCPDS-01-086-0550). In addition, the XRD pattern does not show any diffraction peaks related to other phases β, γ, or δ–Fe_2_O_3_. Furthermore, no peaks were assigned significantly to the Ag which might be attributed to the small Ag content in the prepared samples. Another reason may be due to the complete doping process of Ag into the host lattice, i.e., a diffusion of ions into the host or a migration of ions to the surface. Since the ionic radius of Ag (1.15 Å) is notably higher than that of the corresponding Fe^3+^(0.635 Å), it is therefore reasonable to consider the migration of Ag particles onto the surface of α–Fe_2_O_3_ [35].

**Fig. 2:**
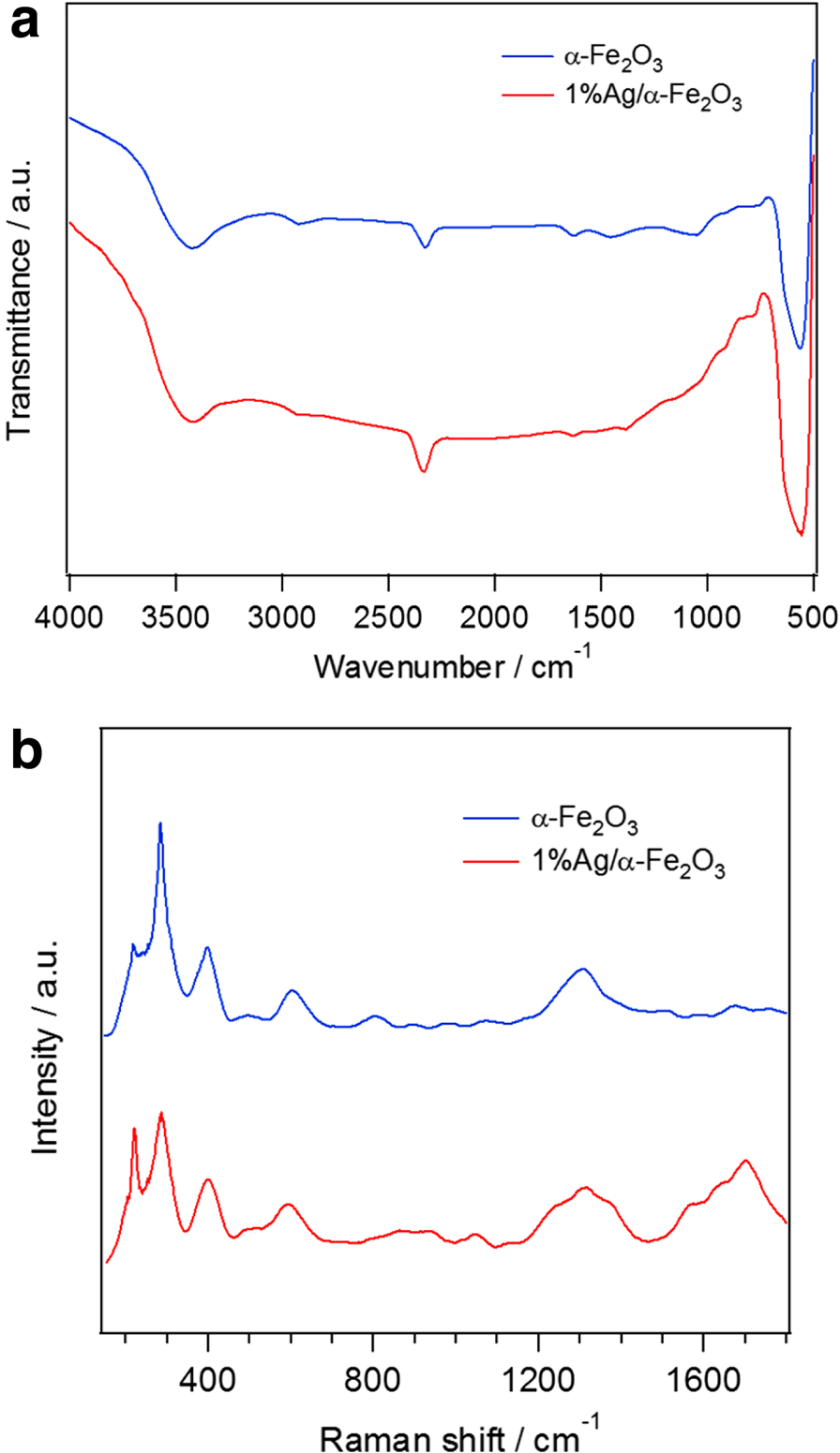
**a** FTIR and **b** Raman spectra of α−Fe_2_O_3_ and 1%Ag/α−Fe_2_O_3_

The presence of functional groups adsorbed on the surface of the synthesized α–Fe_2_O_3_ particles can be examined by Fourier transform infrared (FTIR) spectroscopy. As shown in Fig. 2a, the observed band at ~ 3350 cm^− 1^ with a small one at ~ 1630 cm^− 1^ are assigned to the stretching vibration of water molecules, indicating the existence of a little water adsorbed on the sample. The low frequency band at ~ 566 cm^− 1^ refers to the Fe–O deformation in the octahedral and tetrahedral sites of hematite, giving further evidence for the formation of α–Fe_2_O_3_ in good agreement with the above XRD results. The weak peak at 2900 cm^− 1^ is related to the C–H stretching band, which means some organic compounds are not completely removed from the samples after calcinations [40–42]. Chen et al. [43] prepared hexagonal α–Fe_2_O_3_ nanostructures by a facile alcohol-thermal reaction. They observed wide bands at 3413 cm^− 1^ and weak band at ~ 2900 cm^− 1^, assigned to stretching vibrations of –OH and C–H modes, respectively. Two weak peaks at 1629 and 1420 cm^− 1^ corresponding to asymmetrical and symmetrical vibration of carboxylate groups, indicates a chemical coordination of oxygen atom in acetate anions to iron atoms in unidentate mode [43]. In addition, they observed strong and broad absorptions in the range of 400–700 cm^− 1^ (440, 530, 570, and 650 cm^− 1^). These absorption bands originated from the inherent lattice vibrations of α–Fe_2_O_3_ [43], in good agreement with the present work. On the other hand, Tang et al. [44] demonstrated a novel approach toward development of advanced immune-sensors based on chemically functionalized core-shell Fe_3_O_4_@Ag magnetic nanoparticles. FTIR spectrum of pure Fe_3_O_4_ showed the stretching vibrational modes for the Fe–O bond at 423 and 572 cm^− 1^, whereas for the Ag coated Fe_3_O_4_ the peak at 572 cm^− 1^ shifted to 589 cm^− 1^ and the peak at 423 cm^− 1^ disappeared completely, indicating the coating of Fe_3_O_4_ particles by Ag.

Raman spectra of un-doped and Ag-doped α–Fe_2_O_3_ samples are shown in Fig. 2b. The characteristic spectral peaks of pure α–Fe_2_O_3_ appear at 221, 290, 405, 495, 609, and 1315 cm^− 1^. The peaks located at 221 and 495 cm^− 1^ correspond to the A_1g_ mode and the peaks at 290, 410, and 611 cm^− 1^ are attributed to the E_g_ mode [43–45]. Generally, α–Fe_2_O_3_ belongs to the ${D}_{3d}^6$ crystal space group with seven Raman-active vibration modes, two A_1g_ modes (225 and 498 cm^− 1^), and five E_g_ modes (247, 293, 299, 412, and 613 cm^− 1^) [45], in good agreement with the present work. The sharp peak appears at ~1315 cm^− 1^ is related to a two magnons scattering which arise from the interaction of two magnons created on antiparallel close spin sites [43, 46]. Bhushan et al. [46] observed four more Raman peaks at 666, 820, 1050, and 1103 cm^− 1^ only in highly crystallined α–Fe_2_O_3_ at high Ag-doped α–Fe_2_O_3_. The present work exhibits some of these peaks, confirming the high crystalline nature of the prepared samples. Small degree of Raman shift was observed in Fig. 2b which may be attributed to the differences in both morphology and size of the particles and/or stress. The confirmation of Ag nanoparticles in case of 1%Ag/α–Fe_2_O_3_ sample is evidenced by the bands located at 1370 and 1683 cm^− 1^ [47, 48]. The intensities of Raman peaks of α–Fe_2_O_3_ is less than the relative intensities of the Raman peaks of 1%Ag/α–Fe_2_O_3_ which may be explained by the electric field (EF) enhancement induced by localized surface plasmon resonance (SPR) of the Ag nanoparticles [49]. The electromagnetic effect (EME) associated with large local EF due to the excitation of SPR of Ag and a chemical effect (CE) of the electronic interaction between Ag and α–Fe_2_O_3_ are considered as two essentially different mechanisms control in the surface-enhanced Raman scattering (SERS) phenomenon. The EM contribution is understood to be several orders of magnitude more than the value for the chemical enhancement, and the SPR is fundamentally localized surface plasmon, in contrast to the surface plasmons propagating along the Ag surface. Consequently, the SPR of Ag microstructures plays a main role in the enhancement effect of SERS [50].

**Fig. 3:**
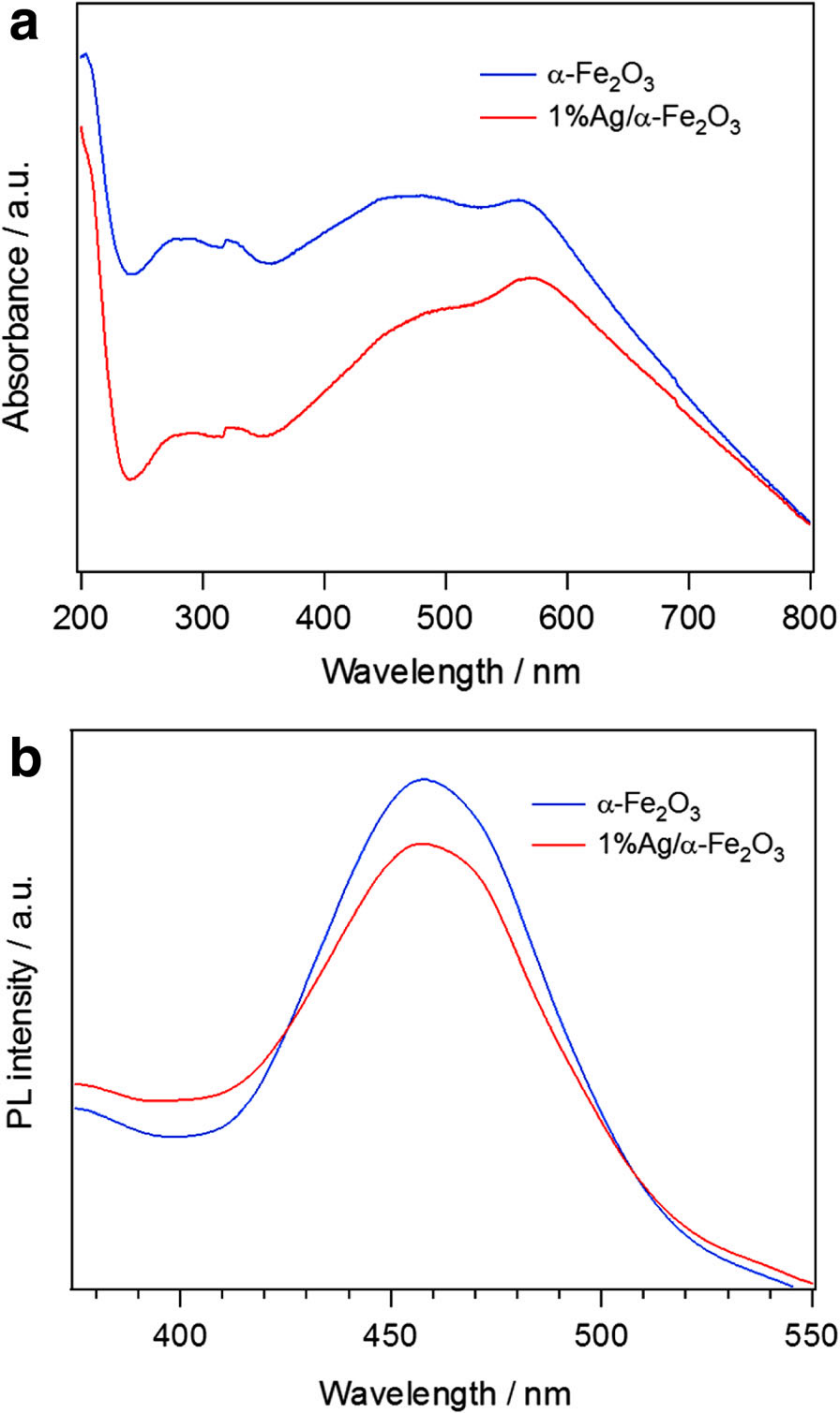
**a** UV-vis optical absorption spectra measured in DI water and **b** PL spectra measured at an excitation wavelength 315 nm for α−Fe_2_O_3_ and 1%Ag/α−Fe_2_O_3_

Figure 3a shows the UV-vis spectra of α–Fe_2_O_3_ and 1%Ag/α–Fe_2_O_3_ samples. In the ultraviolet region (200–400 nm), a two absorbance peaks at around 270–320 nm are observed. The first one is related to the electron transmission of Fe–O in the mechanism of the contribution of the direct charge transition of O_2_^−^ 2p → Fe^3+^ 3d, and the second one may be due to the change in shape and size of the particles [51]. In the visible region (400–800 nm), the narrow absorbance at around 560 nm originates from the indirect charge transition of Fe^3+^ 3d → 3d [52, 53]. In addition, the shift in the broad absorbance peak from 424 to 450 nm peak is due to the surface plasmonic resonance effect of the Ag nanoparticles, i.e., it indicates the presence of Ag nanoparticles on the α–Fe_2_O_3_ [54]. The intensities of absorbance peaks of pure α − Fe_2_O_3_ is higher than 1%Ag/α–Fe_2_O_3_ sample, which is probably due to a decrease in Fe–O resonance; the adsorption of oxygen on Ag surfaces might lead to the formation of surface oxide and may form Fe–Ag interactive species in the hybrid sample [55]. Zhou et al. [51] studied the optical properties of Fe_2_O_3_ thin film synthesized by a modified sol-gel technique. The optical transmittance spectra of the Fe_2_O_3_ film showed a shoulder at 500 nm and a peak at 400 nm. The shoulder peak is assigned to the transition of the 3d non-bonding electrons of the Fe^3 +^ ions to the conduction band in well agreement with the present work, whereas the peak is ascribed to the transition of the 2p bonding electrons of the O^2 –^ ions to the conduction band [51].

With an objective to investigate the recombination processes of the photo-induced electron-hole pairs, the photoluminescence (PL) spectral analysis is employed. The PL spectra of pure α–Fe_2_O_3_ and 1%Ag/α–Fe_2_O_3_ hybrid structure are shown in Fig. 3b. The PL spectra show unique emission bands at wavelength of 460 nm for both α–Fe_2_O_3_ and 1%Ag/α–Fe_2_O_3_. The intensity of this peak is noticeably decreased with Ag-doped α–Fe_2_O_3_ sample in good agreement with the above Raman peak, indicating a lower recombination rate of the photogenerated electron-hole pairs on the Ag/α–Fe_2_O_3_ due to the strong electron-transfer ability of the Ag nanoparticles [55–58]. Kamali et al. [59] observed two PL peaks; the first one located at 710 nm and is a broad and intense. The second one is a shoulder peak at 590 nm. They suggested that these peaks are due to the band-edge emission of the α–Fe_2_O_3_ nanoparticles [59]. Recently, the PL emission peaks at 532, 567, 646, and 697 nm observed by Thomas et al. [60]. These peaks related to different optical band edges due to quantum confinement effect.

**Fig. 4:**
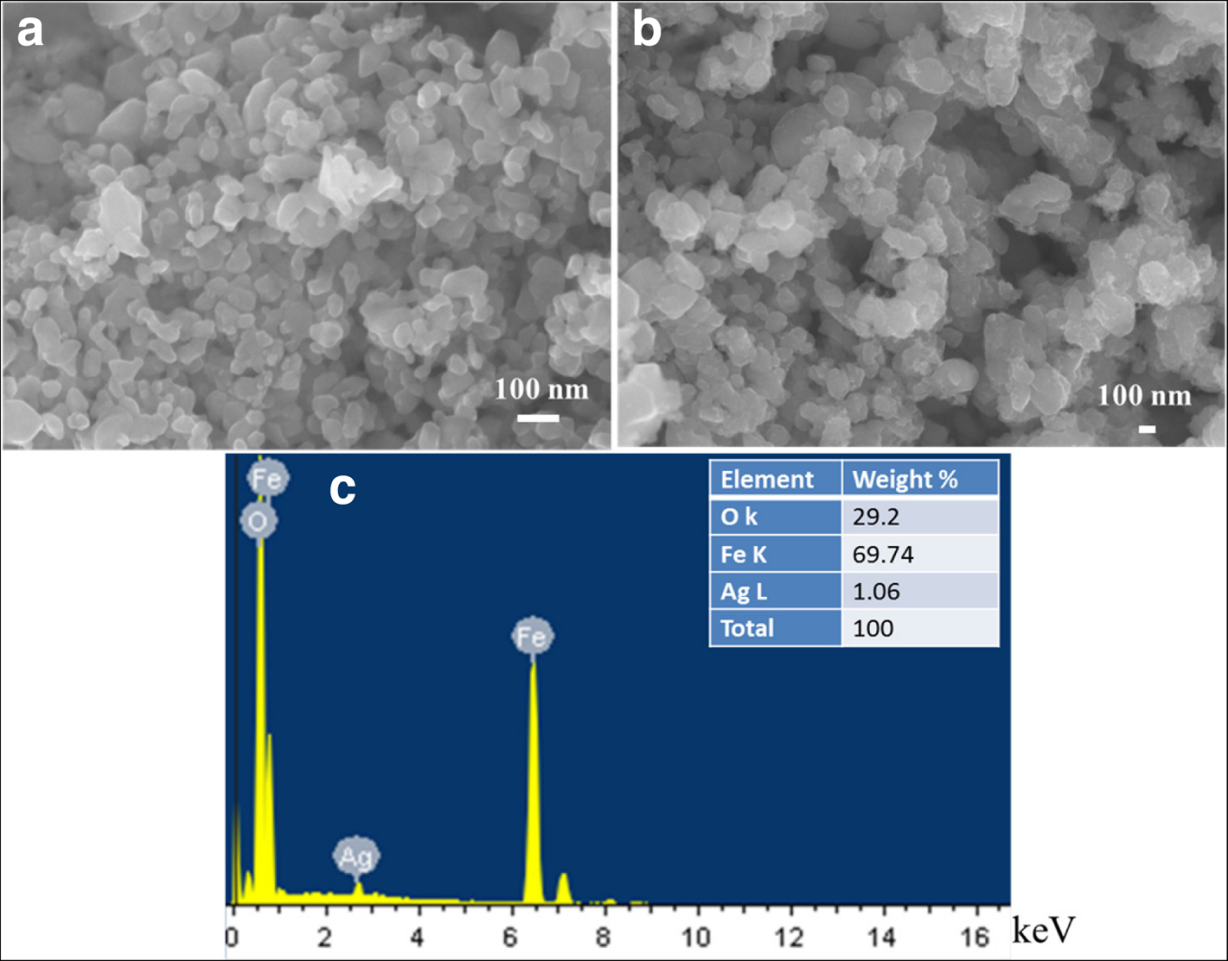
SEM images of **a** α−Fe_2_O_3_, **b** 1%Ag/α−Fe_2_O_3_, and **c** EDS analysis of 1%Ag/α−Fe_2_O_3_ sample

Figure 4 shows the morphology of the prepared α–Fe_2_O_3_ and 1%Ag/α − Fe_2_O_3_ hybrid structure in addition to the corresponding EDS chemical analysis. As could be seen, pure α − Fe_2_O_3_ sample, image (a), exhibits semi-spherical like morphology with the particle size in the range of 25–70 nm. Furthermore, no considerable modification in the particle shape has been attained due to the incorporation of the Ag nanoparticles; SEM image (b). The EDS spectral pattern confirmed the presence of Ag nanoparticles in the developed hybrid nanostructures, with the Ag loading content that matched well with the experiment.

**Fig. 5:**
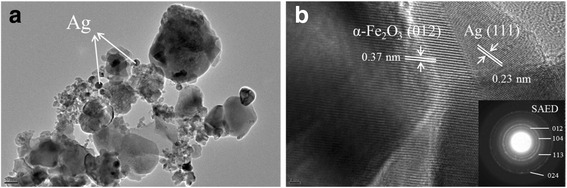
TEM image of **a** 1%Ag/α−Fe_2_O_3_ and **b** the corresponding HR-TEM image with SAED pattern as an inset

Detailed morphological analysis was performed using TEM. Figure 5 presents the TEM image of 1%Ag/α − Fe_2_O_3_ and the corresponding HR-TEM image with the selected area electron diffraction (SAED). TEM image (a) affirmed the attack of Ag nanoparticles to the surface of the host Fe_2_O_3_ matrix, with particle sizes < 20 nm. The main α−Fe_2_O_3_ matrix revealed very fine spherical nanoparticles in the range of 10–30 nm, with some larger spheres forming a shell like structure and collecting those small nanoparticles inside. The HR-TEM image (b) of the prepared doped sample revealed clearly the lattice fringes of α−Fe_2_O_3_ matrix, along with that corresponding to the Ag nanoparticles. The measured inter-planar spaces are 0.37 and 0.23 nm corresponding respectively to the (012) and (111) planes of hexagonal α−Fe_2_O_3_ lattice and cubic Ag, confirming again the presence of Ag in the synthesized hybrid nanostructure. As revealed from the SAED, inset of image (b), the diffraction patterns show different planes of hexagonal cubic α−Fe_2_O_3_ of 012, 104, 113, and 024 corresponding to *d* values of 3.73, 2.70, 2.24, and 1.81 Å, respectively.

**Fig. 6:**
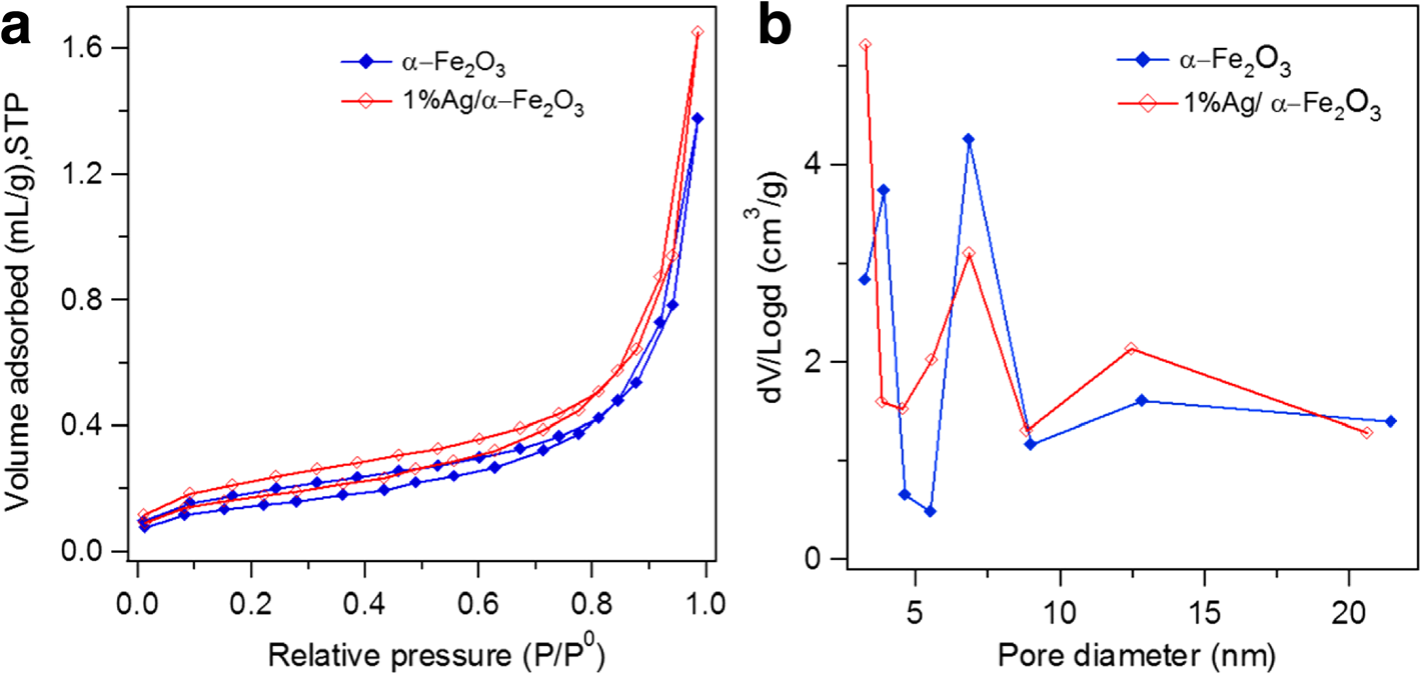
**a** N_2_ sorption isotherms and **b** BJH pore size distribution plots of α−Fe_2_O_3_ and 1%Ag/α−Fe_2_O_3_

**Fig. 7:**
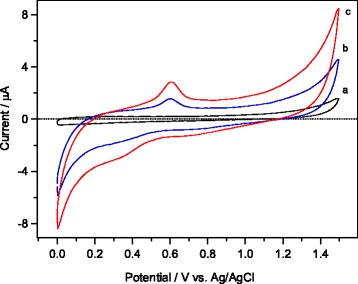
Cyclic voltammograms measured in 0.1 M PBS (pH 7) at a scan rate 50 mVs^− 1^ in the presence of 5 mM ethanol on **a** bare GCE, **b** mesoporous Fe_2_O_3_-modified GCE ,and **c** mesoporous 1 wt.% Ag/Fe_2_O_3_-modified GCE

N_2_ adsorption–desorption isotherm at 77 K was measured to examine the textural properties of the synthesized materials as shown in Fig. 6a. As revealed, both α–Fe_2_O_3_ and Ag/α–Fe_2_O_3_ showed typical type IV profile with H1 hysteresis loop, corresponding to cylindrical pore geometry with high uniformity in pore size and facile pore connectivity [61]. A sharp increase in adsorption volume of adsorbed N_2_ was detected at P/P0 larger than 0.8, which is essentially associated with capillary condensation, indicating sample homogeneity and small pore sizes. The specific surface area and total pore volume of α–Fe_2_O_3_ are 3.55 m^2^/g and 0.004 cm^3^/g, respectively, while the corresponding values for 1%Ag/α–Fe_2_O_3_ are 3.74 m^2^/g and 0.006 cm^3^/g. As can be noticed, a negligible change in textural characteristics was achieved after Ag deposition. Additionally, the pore size distribution is shown Fig. 7b. The α–Fe_2_O_3_ possesses multiple pore sizes with dominant pores at 8 nm along with other minor pores at 4 and 13 nm. The major pore size at 8 nm may be related to the pores initially formed by Pluronic F-127 triblock co-polymer template. Quite similar pore size distribution was observed after Ag deposition, except the major pores are detected at ~ 4 nm probably due to the formation of Ag nanoclusters.

### Electrochemical Behavior of Modified Electrodes

To understand the electrocatalytic behavior of the working electrodes, cyclic voltammetry (CV) technique was firstly applied in a buffer solution of 0.1 M PBS (pH 7) at a scan rate 50 mVs^− 1^ on bare GCE, mesoporous α–Fe_2_O_3_-modified GCE, and mesoporous 1 wt.%Ag/α–Fe_2_O_3_-modified GCE using a fixed concentration of 5 mM ethanol. The CV curves are shown in Fig. 7. As revealed from the CV graph of Fig. 7a, a small anodic current was detected in case of using bare GCE. Meanwhile, significant increase in anodic currents was observed at both mesoporous α–Fe_2_O_3_-modified GCE (graph b) and mesoporous 1 wt.%Ag/α–Fe_2_O_3_-modified GCE (graph c) in comparison to bare GCE (graph a), indicating enhanced electrocatalytic activity of the modified electrodes. To compare both modified electrodes, one noted a maximum anodic current of (*I* = 4.5 μA, graph b) for pure α–Fe_2_O_3_-modified GCE, whereas the 1 wt.%Ag/α–Fe_2_O_3_-modified GCE (graph c) typically generated maximum current (*I* = 8.4 μA), about two-fold current more than pure α–Fe_2_O_3_-modified GCE. In addition, during the reverse scan, the cathodic current is likely attributed to the reduction of water, and those current values were found to increase in the order of 1 wt.%Ag/α–Fe_2_O_3_ > pure α–Fe_2_O_3_ > bare GCE. The noticeable increase in the anodic current suggests a faster electron transfer reaction, and thus allowing efficient detection of ethanol via the oxidation at the 1 wt.%Ag/α–Fe_2_O_3_-modified GCE.

**Fig. 8:**
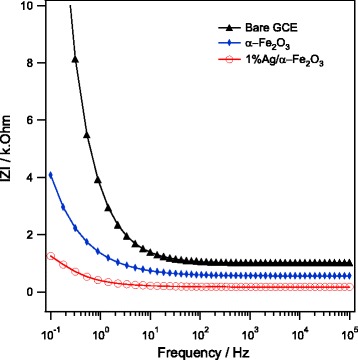
EIS bode plots measured in 0.1 M PBS using bare GCE, α−Fe_2_O_3_, and 1%Ag/α−Fe_2_O_3_-modified GCEs at 5 mV potential amplitude, 0.0 V vs. Ag/AgCl in a frequency range of 0.1 Hz–100 kHz

The electrochemical impedance spectroscopy (EIS) was then employed to investigate the interfacial properties of modified electrodes. Bode plots recorded within the frequency range (0.1 Hz–100 kHz) in PBS solution using bare GCE, α–Fe_2_O_3_, and Ag/α–Fe_2_O_3_-modified GCEs are shown in Fig. 8. Compared to either α–Fe_2_O_3_ or Ag/α–Fe_2_O_3_-modified GCEs, bare, unmodified GCE exhibits relatively larger impedance response. A reduction in impedance at both modified electrodes was detected, indicating an enhanced electrochemical activity. The lowest impedance with higher tendency for electron transfer process is obtained in case of Ag-doped α–Fe_2_O_3_-modified electrode.

### Electrochemical Sensing of Ethanol at Ag/α–Fe_2_O_3_-Modified GCE

**Fig. 9:**
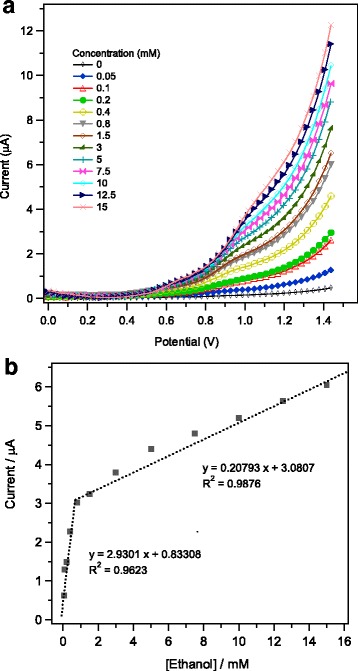
**a** Typical *I-V* characteristics of mesoporous 1wt.%Ag/Fe_2_O_3_-modified GCE toward various concentrations of ethanol (from 0.05 to 15 mM), measured in 0.1 M PBS solution (pH = 7) and **b** the corresponding calibration plot

A simple current-potential (*I-V)* technique is employed here to examine and evaluate the electrochemical sensing behavior of ethanol at the modified active electrodes. The *I-V* responses measured on 1wt.%Ag/α–Fe_2_O_3_-modified GCEs at 50 mVs^− 1^ in 0.1 M PBS (pH 7) using various concentrations of ethanol (0.05 to 15 mM) are collected in Fig. 9a. As could be seen, the anodic current gradually increased with increasing ethanol concentration. Such electrochemical behavior can be related to the increase in the ionic strength of the electrolytic PBS buffer solution with the concentration of ethanol [62]. More ions in solution could provide more electrons to the electrode surface, leading to enhanced conductivity of 1wt.%Ag/α–Fe_2_O_3_-modified electrodes [63]. In other words, at higher ethanol concentration, larger extent of chemi-sorption of ethanol molecules is expected, which in turn led to considerable change in the electronic states at the electrode-electrolyte interface, and thus the current response is enhanced [64]. From the data of the above (*I-V*) response Fig. 9a, the calibration plot was calculated using the average current values and the obtained result is shown in Fig. 9b. As revealed, the calibration plot displays two different slopes related to two linear zones. Such different linear zones correspond to two different ranges of ethanol concentrations: (i) lower concentration from 0.05 to 0.8 mM and (ii) higher concentration from 0.8 to 15 mM ethanol. For higher ethanol concentration > 0.8 mM, the anodic current exhibits a linear behavior with ethanol concentration but with appreciable decrease in sensitivity (the slope of linear zone). The sensitivity decline observed at higher ethanol concentration is likely related to the saturation of the electrode active sites with ethanol target molecules. For both concentration zones, two fitted linear Eqs. (1) and (2) could be generated as follows:

1 $$ \mathrm{at}\ \mathrm{lower}\ \mathrm{concentration}\ \left({R}^2=0.9623\right):\kern3em I\left(\upmu \mathrm{A}\right)=2.9301\ \left[\mathrm{ethanol}\right]\ \left(\upmu \mathrm{A}\right)+0.83308 $$

2 $$ \mathrm{at}\ \mathrm{higher}\ \mathrm{concentration}\ \left({R}^2=0.9876\right):\kern3em I\left(\upmu \mathrm{A}\right)=0.20793\ \left[\mathrm{ethanol}\right]\ \left(\upmu \mathrm{A}\right)+3.0807 $$

The sensitivity of the Ag/α–Fe_2_O_3_-modified GCE was then calculated from the ratio of the slope of the calibration plots, Fig. 9b, and the active surface area of working electrode; the sensitivity values were found to be 41.27 μAmM^− 1^ cm^− 2^ at the lower ethanol concentration zone and 2.93 μAmM^− 1^ cm^− 2^ at the higher ethanol concentration zone. It is worthy to mention that similar research findings of a two sensitivity regions (two different slopes) at different concentrations have been previously observed for ethanol detection using a polypropylene carbonate/silica-modified electrode [65] and for the Pd/ZnO nanocomposite-modified GCE [66]. It has been postulated that the phenomenon of two sensitivity regions can be explained according to the different adsorption modes of ethanol onto the sensor surface; a physisorption process occurs at the lower concentration region leading to higher sensor sensitivity and a chemisorption mode takes place within the higher concentration region giving a saturation to the sensor surface and consequently reducing the sensitivity [65]. Such a two different linear zones obtained with different sensitivities have been also recognized during the electrochemical detection of hydrazine on modified GCE and was discussed in terms of changes in diffusion coefficient of hydrazine due to the evolution of nitrogen gas at higher concentration of target molecule [67]. In the current sensor-modified electrode with Ag/α–Fe_2_O_3_, it was observed that by increasing the ethanol concentration above 15 mM, a saturation of recorded anodic current is achieved, leading finally to a sensing limitation region. The limit of detection (LOD) using the current sensor design was estimated by applying the below Eq. (3) [68], taking into consideration the signal-to-noise ratio of (*S*/*N* = 3).

3 $$ \mathrm{LOD}=3{S}_b/m $$

As indicated above in (Eq. 1), the slop of the calibration graph at lower concentration zone *m* = 2.9301 μAmM^− 1^ and the value of (*S*_*b*_ = 0.015 μA) is the standard deviation calculated for a blank sample after five current measurements. The LOD is accordingly estimated as 15.4 μM.

With an objective to examine the sensing response of current modified electrode toward other alcohols, similar *I-V* experiments have been conducted for both methanol and isopropanol in liquid phase. Table 1 collects the average oxidation currents in microampere, along with the estimated electrode sensitivity in μAmM^− 1^ cm^− 2^ using different alcoholic solutions at 0.05, 0.1, 0.2, and 0.8 mM concentrations. As revealed, the Ag/α–Fe_2_O_3_-modified electrode exhibits the highest current response and sensitivity toward ethanol compared to other two-tested alcohols. The order of sensor response is ethanol > methanol > isopropanol.

**Table 1:** Average oxidation current and electrode sensitivity for different alcohols using Ag/α–Fe_2_O_3_-modified GCE

Alcohols	Current (μA)				Sensitivity (μAmM^− 1^ cm^− 2^)
	0.05 mM	0.1 mM	0.2 mM	0.8 mM	
Ethanol	0.625	1.29	1.48	3.03	41.27
Methanol	0.56	1.16	1.33	2.73	36.28
Isopropanol	0.43	0.90	1.04	2.12	28.20

**Fig. 10:**
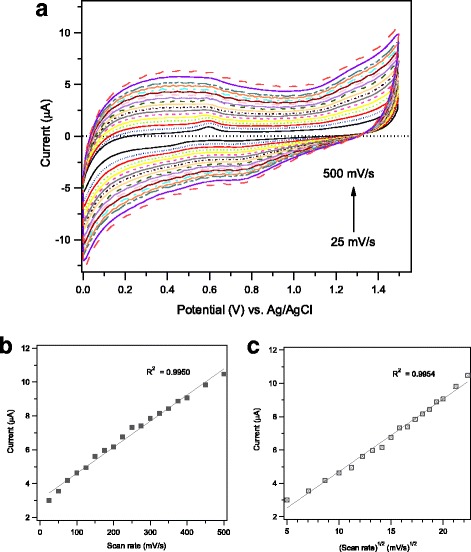
**a** Cyclic voltammograms of mesoporous 1wt.%Ag/Fe_2_O_3_-modified GCE measured in 0.1 M PBS solution (pH = 7) containing 0.2 mM ethanol at various scan rates of 25, 50, 75, 100, 125, 150, 175, 200, 225, 250, 275, 300, 325, 350, 375, 400, 450, and 500 mV/s. Plot of anodic peak current versus scan rate (**b**) and versus square root of scan rate (**c**)

The kinetics of the electrochemical reaction taking place at the electrode surface during ethanol detection was further investigated by cyclic voltammetry technique through the variation of the potential scan rate within the range (25–500 mV/s) and measuring the corresponding anodic currents. Figure 10a collects the cyclic voltammograms recorded at the Ag/α–Fe_2_O_3_-modified GCE in 0.1 M PBS solution (pH = 7) containing 0.2 mM ethanol at various scan rates of 25, 50, 75, 100, 125, 150, 175, 200, 225, 250, 275, 300, 325, 350, 375, 400, 450, and 500 mV/s. As could be revealed, a gradual increase in the anodic peak currents with the scan rate is notably detected, simultaneously in the reverse scan direction, the cathodic currents increase also with the scan rate. Figure 10b exhibits a good linear relation between the anodic peak currents and the scan rate, with a correlation coefficient (*R*^2^ = 0.9950), indicating a surface-controlled kinetic process. Furthermore, in Fig. 10c, the peak currents show a linear dependence on the square root of the scan rate giving *R*^2^ = 0.9954, which is a characteristic feature for a diffusion-controlled reaction. Such kinetics study suggests that the oxidation of ethanol on the current mesoporous 1wt.%Ag/α–Fe_2_O_3_-modified GCE likely proceeds via a mixed surface reaction and diffusion-controlled kinetics.

Table 2 shows a comparison of previously reported results of various modified electrodes during the ethanol sensing using the *I-V* technique. The sensitivity observed herein using the current sensor electrode is significantly higher as compared to the recently reported sensitivities particularly at the lower concentration regime [62, 65, 66, 69–75].

**Table 2:** Comparison of sensing performance of ethanol with previously reported modified electrodes

Modified electrode	Linear range (mM)	LOD (mM)	Sensitivity (μAmM^−1^ cm^−2^)	Ref.
SnO_2_–ZnO	0.195–25	0.137	62.56	[62]
Polypropylene carbonate/silica	0.17–850	0.021	0.5698	[65]
Mesoporous Pd–ZnO	0.05–0.8	0.0192	33.08	[66]
Gd_2_O_3_ nanostructures	0.17–850	0.052	0.266	[69]
ZnO–CeO_2_	0.17–1700	0.16	0.8331	[70]
CuO nanosheets	0.17–1700	0.143	0.9722	[71]
Ni-doped SnO_2_	10^−6^–1	0.6 × 10^− 6^	2.3148	[72]
Mg(OH)_2_ nanodisks	10^−4^–10	73 × 10^− 6^	6.89	[73]
poly(1–naphthylamine)	0.78–50	–	1.66	[74]
Mg(OH)_2_ nanosheets	0.01–1000	0.005	3.991	[75]
Mesoporous Ag/α–Fe_2_O_3_	0.05–0.8	0.0154	41.27	This work
	0.8–15		2.93	This work

An important piece of information remains regarding how the sensing mechanism would proceed in the current modified electrode based-system. In general, it has been proposed that the chemisorbed oxygen species (O^−^, O_2_^−^, or O_2_^2−^) will cover the surface of the modified electrode [76]. A space-charge region is accordingly originated via electrons withdraw from the surface of sensor electrode. A surface reaction between oxygen species and adsorbed ethanol molecules takes place, releasing electrons to the conduction band of α–Fe_2_O_3_ material, Eq. (4) [72], and thus the conductivity and sensor response were enhanced.

4 $$ {\mathrm{C}}_2{\mathrm{H}}_5\hbox{--} {\mathrm{O}\mathrm{H}}_{\left(\mathrm{ads}.\right)}+6\ {{\mathrm{O}}^{\hbox{--}}}_{\left(\mathrm{ads}.\right)}=2{\mathrm{C}\mathrm{O}}_2+3{\mathrm{H}}_2\mathrm{O}+6{e}^{\hbox{--} } $$

The metallic Ag and metal oxide α–Fe_2_O_3_ would have different surface catalytic active sites with electrochemical behavior that would promote the adsorption and diffusion processes of ethanol molecules onto the working electrode. Therefore, the superior sensing performance obtained here with the newly developed mesoporous Ag/α–Fe_2_O_3_-modified GCE is likely related to the mesoporosity of α–Fe_2_O_3_, small particle size of Ag nanoparticles with catalytic function, chemical, and electronic sensitization effect, all of which would provide enormous adsorption sites for ethanol molecules and promote the diffusion process. Via doping the α–Fe_2_O_3_ by Ag nanoparticles, the current sensor-based-modified electrode exhibited extremely high sensitivity toward ethanol detection as 41.27 μAmM^− 1^ cm^− 2^ with a very low LOD of 15.4 μM at (S/*N* = 3) at room temperature.

For the sake of sensor practicability, the storage and operational stability along with repeatability, and reproducibility of modified electrodes were evaluated. Using three different active, modified GCEs, the cyclic voltammograms recorded in 5 mM ethanol gave a relative standard deviation (RSD) ~ 4%, which implies good reproducibility. Five successive cyclic tests in the same ethanol solution yielded < 5% RSD, indicating good electrode repeatability. A proper operational stability of the modified electrode was observed during its continuous testing for 45 min in ethanol solution with a minor reduction in current response. Finally, no special care is required for electrode storage; the present Ag/α–Fe_2_O_3_-modified GCE showed unique storage stability for 5 weeks with almost no surface deterioration or reduction in sensitivity.

## Conclusions

In summary, an efficient ethanol electrochemical sensor based on mesoporous Ag/α–Fe_2_O_3_ synthesized by a facile sol-gel and photo-reduction procedures has been described. The mesoporous α–Fe_2_O_3_-modified GCE exhibited good electrocatalytic activity during the detection of ethanol in phosphate buffer solutions. Doping the active material α–Fe_2_O_3_ by Ag nanoparticles led to superior sensing performance at room temperature. An extremely high sensitivity of 41.27 μAmM^− 1^ cm^− 2^ at low ethanol concentration (0.05 to 0.8 mM) with a very low LOD 15.4 μM at (*S/N* = 3) was obtained. Additionally, the sensing response and electrode sensitivity was found to be much higher for ethanol as comparted to either methanol or isopropanol. Such extraordinary sensing performance was likely related to mesoporosity of α–Fe_2_O_3_ matrix, along with the small particle size of Ag nanoparticles. The unique sensing characteristics obtained in this study reveal that the current-developed mesoporous Ag/α–Fe_2_O_3_ would represent a potential sensing material for further fabricating high-performance electrochemical sensors for the detection of ethanol or similar alcohols in aqueous solutions.

## Abbreviations

Ag: Silver
*CV*: Cyclic voltammetry
EIS: Electrochemical impedance spectroscopy
F-127: Pluronic triblock copolymer
GCE: Glassy carbon electrode
*I-V*: Current versus potential
LOD: Limit of detection
*m*: Slop of the calibration graph
PBS: Phosphate buffer solution
*R*^2^: Correlation coefficient
*S*/*N*: Signal-to-noise ratio
*S*_*b*_: Standard deviation
α–Fe_2_O_3_: Hematite (iron oxide)
